# Assessment of personal care and medical robots from older adults’ perspective

**DOI:** 10.1186/s40638-017-0061-7

**Published:** 2017-09-20

**Authors:** K. M. Goher, N. Mansouri, S. O. Fadlallah

**Affiliations:** 1 School of Life & Health Sciences, Aston University, Aston Triangle, Birmingham, B4 7ET UK; 2 Department of Land Management and Systems, Faculty of Agribusiness and Commerce, Lincoln University, PO Box 85084, Lincoln 7647, Christchurch, New Zealand; 3 Department of Mechanical Engineering, Auckland University of Technology, 55 Wellesley St, Auckland, 1010 New Zealand

**Keywords:** Assistive medical robots and devices, Robot assessment, Older adults’ perspective, Assistive walking devices, Information and communication technology, Older adults’ needs assessment

## Abstract

Demographic reports indicate that population of older adults is growing significantly over the world and in particular in developed nations. Consequently, there are a noticeable number of demands for certain services such as health-care systems and assistive medical robots and devices. In today’s world, different types of robots play substantial roles specifically in medical sector to facilitate human life, especially older adults. Assistive medical robots and devices are created in various designs to fulfill specific needs of older adults. Though medical robots are utilized widely by senior citizens, it is dramatic to find out into what extent assistive robots satisfy their needs and expectations. This paper reviews various assessments of assistive medical robots from older adults’ perspectives with the purpose of identifying senior citizen’s needs, expectations, and preferences. On the other hand, these kinds of assessments inform robot designers, developers, and programmers to come up with robots fulfilling elderly’s needs while improving their life quality.

## Introduction

Recent decades have witnessed a noticeable development in information and communication technology (ICT). This development has led to advent of various types of robots in vast majority of industries, namely manufacturing, military, medical and health care, entertainment, and household [73]. In the medical sector, assistive medical robots and devices play substantial role in senior citizens lives. The population of senior citizens is growing substantially over the world [5, 48]; therefore, the demand for specific needs rises [13, 52, 53]. Growth in aging population results in noticeable number of issues such as dearth of health-care centers, professionals, and services [30] as well as huge burdens of health-care costs [1]. In order to diminish costs related to readmission and transportation, and also to ameliorate quality of health-care services and older adult’s independency, health-care services are shifted to older adults’ home from medical centers [9]. Therefore, different types of assistive medical robots, namely remote presence robot, paro-robot, telerobot, skillegent robot, RIBA [1], and devices such as wheeled walkers [7, 57], are created to fulfill various needs and compensate disabilities. Assistive medical robots and devices not only have facilitated older adult’s tasks, but also have promoted their life quality and kept their autonomy [56]. For instance, mobile manipulated robot offers to bring object(s) to older adults or by their request [2], telerobot monitors health condition and medication of elderly [1], pet robot companies older adults [6], and rolling walker assists elderly to have better mobility, stability, and balance [68].

### Overview and contribution

There are a noticeable number of assistive robots and devices to empower older adults to carry out their daily routine tasks independently. Yet in accordance with conducted research studies, older adults do not incline toward the use of technology. In other words, there is a gap for improving assistive technology to increase robot acceptance and fulfill elderly’s needs. The authors of this paper provide a review of assessment of assistive medical robots and devices from older adults’ perspective to identify the factors associated with assistive technology acceptance. The authors of this paper believe that adequate and accurate understanding of senior citizens’ needs and expectations will inform robot designers, programmers, and developers to create user-friendly and user-centered robots and devices meeting required features and functions. We aim at identifying the reasons causing decline of robot acceptance and also to assess older adults’ needs and expectations. We believe that in order to boost acceptance of older adults to use robots, it is important to assess not only their needs and expectations, but also their attitudes toward technology.

### Paper organization

This paper is organized as follows: “Assistive technologies overview” section presents a detailed overview of assistive technologies and their associated features. “Assessment of assistive medical robots” section introduces an overview of assessment of medical robots from older adults’ perspectives. In “Assessment of walking devices and related technologies” section, we investigate the assessment of assistive walking aids and in particular walking devices.

“Older adults satisfaction of other assistive devices” section focuses on presenting older adults’ satisfaction of other assistive devices. The paper is concluded in “Conclusion” section where we emphasize on specific attitudes of older adults toward the use of assistive technologies in daily life.

## Assistive technologies overview

Different types of robots have been developed to provide various aids for older adults. The information in Table 1 reveals that enhancements in technology have compensated elderly’s disabilities, which improved their life quality and health conditions through remote controlling robots [19]. Moreover, assistive robots and devices are developed to provide physical aid to elderly to accomplish their routine activities such as feeding, management of medication, and emergency control [35, 55]. Besides, it is obvious that older adults benefit from assistive robots and devices to retain their autonomy, diminish health-care needs, accomplish daily tasks, and increase social communication [10]. Albeit a great number of useful assistive robots and devices are developed, yet some older adults decline to accept technology in their routine life [13].

**Table 1:** Assistive medical robots and devices for older adults

Category	Description and primary functions	Research contributions
Telerobots	The functions of this type of robot are to facilitate communication with medical professionals, to monitor injuries, and also to follow up with family members [1]	Pearl and Wakamuru robot [46], robo robot [46], skilligent robot [1], and RIBA [61]
Mobile manipulator robots	Mobile manipulator robots focus on disabled and older adults with the intention of furnishing requested item to either older adult or disabled to satisfy their needs [2]	Mobile manipulator robot [2]
Assistive walking devices	Assistive walking devices are primarily created to compensate older adults’ disabilities, while maintaining better balance, stability, and walking support. They also help in facilitating mobility, maneuvering, walking, standing, sitting, and independency. These devices are enhanced with information and communication technology to detect fall incidents, fall prevention, and also ameliorate alarming system. The enhancement in walking devices reduces waiting time to receive assistance [31]. Furthermore, ICT assists medical professionals and caretakers to monitor fall incidents closely [14]	Rolling walker [68], knee walker [3], crutch [40], and cane [69]
Animal-like robots	Albeit a great number of medical professionals believe that animals have deleterious health consequences such as injuries and infection, a noticeable number of them subscribe to the belief that interaction with animal leads to emotive effects to patients. For this reason, animal robots with the purpose of communicating with and entertaining older adults, ameliorating health condition, and relieving distressing imitate animal behaviors [70]	Paro-robot [71], NeCoRo [11], AIBO [11], bandit [11], and accompany robots [36]
Home health-care robots (HHRs)	When the primary tasks of a robot are associated with home health care, the robot is called home health-care robot. These kinds of robots assist medical specialists to monitor elderly at their houses. HHRs are designed with the purpose of ameliorating autonomy of older adults as well as improving their well-being to alleviate long-term hospitalization in medical centers. Home health-care services consist of substantial services such as professional and physical nursing care, speech treatment, and medical social services [1]	Tele-operated robot [50]
Humanoid robots	This type of robot primarily identifies older adults’ needs and also provides services for both elderlies and their caregivers. The main features of this robot are to provide medication reminder, to detect issues and take action to inform caregiver, manage plans, and assist elderly to take off [44]	iCub robot and nao robot [21]

## Assessment of assistive medical robots

Though a great number of assistive medical robots and devices are developed for older adults, yet there is lack of research studies related to acceptance of assistive technology from older adults and their caretakers’ perspective [35]. We believe that it is important to conduct further research work surrounding this field. The declined acceptance of older adults of assistive technologies is mainly related to the limited knowledge and the embarrassed emotions [27]. Moreover, [17] it is found out that there are two primary factors affecting use of assistive technology: abilities and attitudes. In accordance with conducted ethnographic studies, older adults incline to utilize assistive technology when the dignity and autonomy of them are maintained [26]. Ethnographic studies provided a series of recommendations to robot designers and developers. The recommendations are in terms of robot dimensions which should be fit within elderly’s place, robot interface which should be easy to use, and interaction feature which should meet elderly’s abilities.

### Older adults’ attitude toward health-care robots

There are two primary factors influence adoption of technology by older adults: ease of use and usefulness [22, 33, 62]. Ease of use factor refers to level of older people’s knowledge about assistive technology. Older adults, who are intermediate and familiar with assistive technology, show positive perspectives [13, 25]. Robot usefulness refers to provision of physical assistance and task monitoring such as carrying and picking up a heavy item [13]. The behavior of older adults has proved that elderly decline to utilize assistive robots if their tasks are not found useful [41]. Findings of the aforementioned research studies have shown that robot functionalities, related to nonsocial tasks and robot interaction, are the most influential factors in technology acceptance by the older people [60].

It is stated that older adults commonly refuse to use assistive technology because of being novice at accomplishing tasks with technology [20]. In addition, it is said that older adults, unlike young people, are concerned about learning technology skills. This tends to make them refusing to use technology [23]. From a large-scale research study, it is found that older adults show positive attitudes toward assistive technology adoption when they are assisted with significant task [25]. A number of research studies revealed that cost is one of the primary factors which make older people concerned. They incline to adopt assistive technology if the advantages outweigh the cost [15, 45, 59]. In accordance with previous studies, the use of technology appeals older adults if it only offers them greater autonomy [51, 65]. Moreover, unlike youngsters, older adults show different attitude toward technology acceptance. Older adults decline to trust on technology, and also they think it is complex to utilize [58]. Moreover, the behaviors of older adults have proved that when they face difficulties, they tend to give up rather than asking for help [28].

In other conducted research work by Wu et al. [72], they investigated adoption of assistive robots by elderly and also analyzed elderly’s perspective after 1 month of direct interaction with assistive robots. Two groups of cognitively intact healthy (CIH) and mild cognitive impairment (MCI) participated in this study. Both groups declined to show willingness to utilize assistive robots. Moreover, negative attitudes toward robots and negative image of robots were noticed. The same attitude has been reported after carrying the same study for one more month of interaction. Older people responded that assistive robots are not useful, whereas they found robots safe, interesting, and easy to use. This finding reveals a total contrast with previous studies, indicating that older adults’ behavior toward assistive robots ameliorates after direct interaction [39, 63]. It has been noticed in this study that older people found themselves not in needs of assistive robots.

In the work done by Morris et al. [49] and Heart and Kalderon [32], elderly showed fear of dehumanization toward adoption of assistive robots. Ethical and societal issues were considered as a barrier of adoption of assistive technologies. Participants responded that use of assistive robots gives them the impression of being watched and monitored. This gives rise to exceeding the importance of elderly’s privacy.

Beer and Takayama [10] assessed mobile remote presence (MRP) systems from older adults’ point of view. They reported that benefits of MRP systems were obvious to elderly; therefore, older adults showed willing to utilize such a system in social and medical contexts. Older adults had positive attitudes to number of benefits from assistive robots, namely decreased traveling cost, improved visualization, and reduction in social isolation. On the other hand, they were concerned about call management, lack of face-to-face communication, and privacy.

### Older adults’ preferences from health-care robot’s functions

Older people prefer to have far more communication with health-care robots. For instance, they prefer to converse with robots about the topic related to robot itself, rather than talking about health-care and activities [41]. Moreover, older people consider robots as a performance-directed machine, rather than a social device [25]. Broadbent et al. [13] conducted an important research work to investigate not only older adults’ perspectives toward health-care robots, but also their caretakers as well. In their study, it was found that caregivers were concerned about their jobs that may be replaced by health-care robots. On the other side, this research highlighted that older adults have positive perspective about health-care robot apart from concerns related to reliability, privacy, and safety. In terms of robot’s functionality, fall detection feature appealed vast majority of elderly. Moreover, functions such as big buttons, clear voice, and visible screens are significantly favorable. Older adults prefer robots to automatically detect and monitor fall incidents without wearing any device or being nearby a call button.

Past research work revealed that in terms of robot appearance, unlike youngsters, older adults prefer less human-like and more serious robots [4, 16, 58]. It is stated that the robot’s tasks should be commensurate with appearance and shape. Moreover, the robot is not necessarily required to be human-like if its functions do not require. In terms of size, adjustable robots with minimum of five feet are highly accepted.

Further research work has been conducted by Smarr et al. [60] with the purpose of identifying the tasks that need robot assistance. In this study, tasks were categorized into three categories: self-maintenance activities of daily living (ADLs), instrumental activities for daily living (IADLs), and enhanced activities of daily living (EADLs). Assistance for IADL tasks consists of housekeeping such as laundry and medication reminder. On the other hand, tasks such as new learning and pastime refer to EADL. Older people prefer to have robot assistance rather than human assistance for IADLs and then EADLs. In contrast, it was found that older people favor to have assistance for ADLs and also some specific tasks of IADLs and EADLs, namely decision on medication, meal preparation, and social interaction. The results of this study are similar to Broadbent et al. [13] findings. This makes us able to conclude that older adults prefer to have robot assistance for monitoring and physical aid, while they prefer human aid for decision-making tasks.

Considering medication management as a prime example, older adults prefer health-care robots to either bring them medicine or remind them of the regular doses. However, they favor human assistance to make decision what and/or when medicine to take. This concept assists designers and developers of health-care robots to furnish robot with high level of intelligent to enable them to make the right decision.

## Assessment of walking devices and related technologies

Wheeled walkers provide walking support for a big number of older people to compensate their moving and walking disabilities. Wheeled walkers are used primarily for maintaining mobility and balance [8, 57] as well as alleviating fall incidents [29]. Though they are used by a noticeable number of users, yet there is a need for improvement to fulfill older adults’ needs and expectations [42]. This section gives a review of previous conducted research studies on the assessment of assistive walking devices from older adults’ perspective.

### Wheeled walkers limitations

Van Riel et al. [67] reported that the use of wheeled walkers usually results in severe fall injuries. Based on previous research by Lindemann et al. [42], there are various limitations associated with the use of wheeled walkers which causes serious fall incidents to older adults including walking backward, downhill and uphill, holding an item when fronting obstacle(s), encountering obstacles such as stairs in public transportation, and walking on uneven surfaces. Older adults encounter difficulties to retain their balance and control to open a door which is in reverse direction of their assistive wheeled walker. This situation becomes more challenging when a user holds an item while passing through a door. For this reason, older adults stated that it is easier to walk through a door or to open the door without wheeled walker. Despite there have been numerous approaches and developments to overcome the mentioned limitations of wheeled walkers, the proposed solutions were not satisfactory. For instance, walking backward through a door using a walker is still a challenge for most users. This is due to the fact that front wheels of the walker provide 360° rotation, whereas the rotation of back wheels is restricted. Rentschler et al. [54] recommended a walker with a rotation feature and intelligent obstacle prevention to overcome those limitations.

## Older adults’ satisfaction of other assistive devices

A noticeable number of research works have been accomplished to evaluate older adults’ experience feedback and satisfaction level from assistive technologies. Privacy is considered to have a significant concern to older people. For instance, they prefer to have faint pictures at their private places of the house (bedrooms) while they do not hesitate to have transparent images in other general areas (dining room and lounge) [43, 47]. Cameras and visual surveillance systems are unfavorable to the older adults [64]. Moreover, disabilities in having control over the assistive device are one of the main reasons that older people decline to adopt ICT [18, 38, 43, 66]. They also prefer having complete control over the assistive device [12, 34, 43]. For instance, older people incline to switch off false alarm by themselves. In addition, cost of assistive device and maintenance charges are of a great concern to older adults. This makes them decline acceptance of expensive assistive devices [23, 24, 47, 64]. One more observation is older people favor attractive and dainty devices created in different colors [37]. Additionally, findings of this research show that it is difficult for them to press the button of device and read the gray color text and background [37]. Older adults encountered less hardship to wear wrist devices; therefore, this type of device design impressed them substantially [37]. Brownsell and Hawley [14] indicate that ICT devices empower elderly to feel independent and safe to take risk.

## Conclusion

All in all, various assistive medical robots and devices are designed and developed for growing population of older adults. Although there are common needs and preferences among different segment of older adults, it should be considered that each segment has its specific needs and preferences. Consequently, it is substantial to develop the right assistive robot or device for them. Apart from needs and preferences of older adults, cost of robot or device is a primary factor in acceptance and adoption. Proper management of production cost and design of a sound sale strategy are of great importance in this regard. Research, discovering needs and preferences of elderly from assistive medical robots and devices has paved the ground for researchers and scholars to design robots and devices fulfilling their needs and expectations. Findings around acceptance of assistive devices from older adults’ perspectives should be on top of the data necessary to inform the design and development process. Furthermore, understanding their attitudes while dealing with, approaching by, or having interaction with assistive robots is of great importance to inform the designers, developers, and programmers.
